# Scaling up data curation using deep learning: An application to literature triage in genomic variation resources

**DOI:** 10.1371/journal.pcbi.1006390

**Published:** 2018-08-13

**Authors:** Kyubum Lee, Maria Livia Famiglietti, Aoife McMahon, Chih-Hsuan Wei, Jacqueline Ann Langdon MacArthur, Sylvain Poux, Lionel Breuza, Alan Bridge, Fiona Cunningham, Ioannis Xenarios, Zhiyong Lu

**Affiliations:** 1 National Center for Biotechnology Information (NCBI), National Library of Medicine (NLM), National Institutes of Health (NIH), Bethesda, Maryland, United States of America; 2 Swiss-Prot Group, SIB Swiss Institute of Bioinformatics, Geneva, Switzerland; 3 European Molecular Biology Laboratory, European Bioinformatics Institute, Wellcome Genome Campus, Hinxton, Cambridge, United Kingdom; 4 Center for Integrative Genomics, University of Lausanne, Lausanne Switzerland; 5 Department of Chemistry and Biochemistry, University of Geneva, Geneva, Switzerland; Case Western Reserve University, UNITED STATES

## Abstract

Manually curating biomedical knowledge from publications is necessary to build a knowledge based service that provides highly precise and organized information to users. The process of retrieving relevant publications for curation, which is also known as document triage, is usually carried out by querying and reading articles in PubMed. However, this query-based method often obtains unsatisfactory precision and recall on the retrieved results, and it is difficult to manually generate optimal queries. To address this, we propose a machine-learning assisted triage method. We collect previously curated publications from two databases UniProtKB/Swiss-Prot and the NHGRI-EBI GWAS Catalog, and used them as a gold-standard dataset for training deep learning models based on convolutional neural networks. We then use the trained models to classify and rank new publications for curation. For evaluation, we apply our method to the real-world manual curation process of UniProtKB/Swiss-Prot and the GWAS Catalog. We demonstrate that our machine-assisted triage method outperforms the current query-based triage methods, improves efficiency, and enriches curated content. Our method achieves a precision 1.81 and 2.99 times higher than that obtained by the current query-based triage methods of UniProtKB/Swiss-Prot and the GWAS Catalog, respectively, without compromising recall. In fact, our method retrieves many additional relevant publications that the query-based method of UniProtKB/Swiss-Prot could not find. As these results show, our machine learning-based method can make the triage process more efficient and is being implemented in production so that human curators can focus on more challenging tasks to improve the quality of knowledge bases.

## Author summary

As the volume of literature on genomic variants continues to grow at an increasing rate, it is becoming more difficult for a curator of a variant knowledge base to keep up with and curate all the published papers. Here, we suggest a deep learning-based literature triage method for genomic variation resources. Our method achieves state-of-the-art performance on the triage task. Moreover, our model does not require any laborious preprocessing or feature engineering steps, which are required for traditional machine learning triage methods. We applied our method to the literature triage process of UniProtKB/Swiss-Prot and the NHGRI-EBI GWAS Catalog for genomic variation by collaborating with the database curators. Both the manual curation teams confirmed that our method achieved higher precision than their previous query-based triage methods without compromising recall. Both results show that our method is more efficient and can replace the traditional query-based triage methods of manually curated databases. Our method can give human curators more time to focus on more challenging tasks such as actual curation as well as the discovery of novel papers/experimental techniques to consider for inclusion.

## Introduction

The question of how genetic variation in a population influences phenotypic variation is of major importance in biology. Naturally occurring genetic variants, both rare and common, can provide insight into disease mechanism and protein function. This understanding, coupled with the recent explosion in next-generation sequencing, has meant a dramatic increase in publications on the subject. This also means that it is now impossible for individual researchers to collect and collate all the variant information that may be relevant to them. To assist researchers, variant information in publications is selected, summarized, organized, and stored in a searchable form in knowledge bases such as UniProtKB/Swiss-Prot [1, 2] and the NHGRI-EBI GWAS Catalog [3] for easier access and greater usability. However, such knowledge bases require domain experts to collect and manually curate high quality information from the literature [4], a highly time consuming and therefore costly process. In addition, these knowledge bases have their own focus and collect information from publications according to their own specific guidelines.

As shown in the study of Baumgartner et al., the manual curation of information for genomic databases is, for the most part, not scalable due to the fast-growing number of publications [5]. To overcome this scalability issue, automated or semi-automated methods can be used [6]. For this purpose, text mining and machine learning tools that support information extraction and annotation have been developed [7–14]. Poux et al. [15] demonstrated that manual curation can be more efficient and scalable with the proper text-mining services and techniques.

As a typical first step in the manual curation process, the document triage process involves identifying publications of interest [16, 17]. On average, three thousand biomedical papers are indexed in PubMed every day. (In 2016, more than 1.1 million publications were indexed in PubMed). Triage is then required to select a subset of relevant publications for curation. As Hirschman et al. [16] explained, triage is usually carried out using pre-defined queries in the PubMed database. The queries are generated using general terms related to topics such as “GWAS” and “Drug screening” or a list of entities (e.g., onco-gene list [18]). Publication date or publication type (e.g., review, book) are also included for a query as needed. However, a triage process using pre-defined queries has several major limitations. Using general topic terms for a query may be futile because publications on a certain topic may not contain the specific terms. For example, if a user wishes to find publications on cancer-related genes, the query should contain specific terms such as “HER2,” “Carcinoma,” “Tumor,” and so on, rather than “cancer-related gene” because many publications on cancer-related genes do not contain the general term “cancer-related gene.” Furthermore, the topic words in a publication may provide only background information and may not be on the main topic of the publication. Also, longstanding databases have their own complex and detailed guidelines for manual curation and it can be difficult to create a PubMed query that fulfills their conditions [19]. For these reasons, query-based triage is limited in retrieving highly precise and complete set of relevant publications for further use, not to mention the process itself is also very labor intensive and time consuming.

To overcome the limitations of the query-based triage and manual curation processes, machine learning-assisted curation research studies have been conducted. Poux et al. [15] used PubTator, a web-based curation support system that assists users in annotating publications in PubMed. Curators can read publications that are highlighted and pre-annotated by automated named-entity recognition tools. Curators can also easily upload and generate their curation collection and save their results with a simple mouse click. In their study, Poux et al. selected only thirteen journals from which to collect protein function information, and ranked the publications of the journals by the number of proteins mentioned in the text. However, other than the thirteen selected journals, there are many journals that have published papers on protein function. Also, prioritizing the publications by the number of proteins may not be the best method because some papers include valuable information on a small number of proteins.

Almeida et al. [20] built a method called mycoSORT using support vector machine (SVM), Naïve Bayes and Logistic Model Trees on the triage task for the mycoCLAP database [21]; however, this required several text preprocessing steps and an extensive feature extraction process which are data/domain dependent. Because of these dependencies, their method is not directly applicable to other types of databases. Their feature extraction process is time consuming, labor intensive, and requires domain knowledge from human experts [6].

In recent years, newly proposed deep learning-based text mining methods have started outperforming traditional machine learning-based methods in various tasks [22–28]. In addition, these deep neural network models do not require intensive feature engineering by domain experts; hence, they can also be easily generalized to other tasks with datasets in different domains. In this paper, we propose to employ convolutional neural network (CNN), a class of deep, feed-forward artificial neural networks, for the identification of publications relevant for variant curation. By comparing the results of our method with those of mycoSORT, the method proposed by Almeida et al. [20], we demonstrate how our deep learning-based classifier performs better than traditional machine learning classifiers, even without feature engineering. For assessing the utility of our approach, we applied our method to two external knowledge bases (UniProtKB/Swiss-Prot and the GWAS Catalog) in real-world circumstances. We compare the performance of our proposed method with that of each of the knowledge base-specific query-based methods to demonstrate that our method can greatly improve the efficiency of the document triage step in these two databases. While the usage of CNNs to classify text documents is not new, the application of deep learning to speed up the real-world triage process for biomedical literature is, to the best of our knowledge.

## Results

In this research, our goal is to improve the triage process by predicting the most suitable publications for each knowledge base in their manual curation. We aim to provide both binary and ranked results with scores for each selected publication.

**Fig 1:**
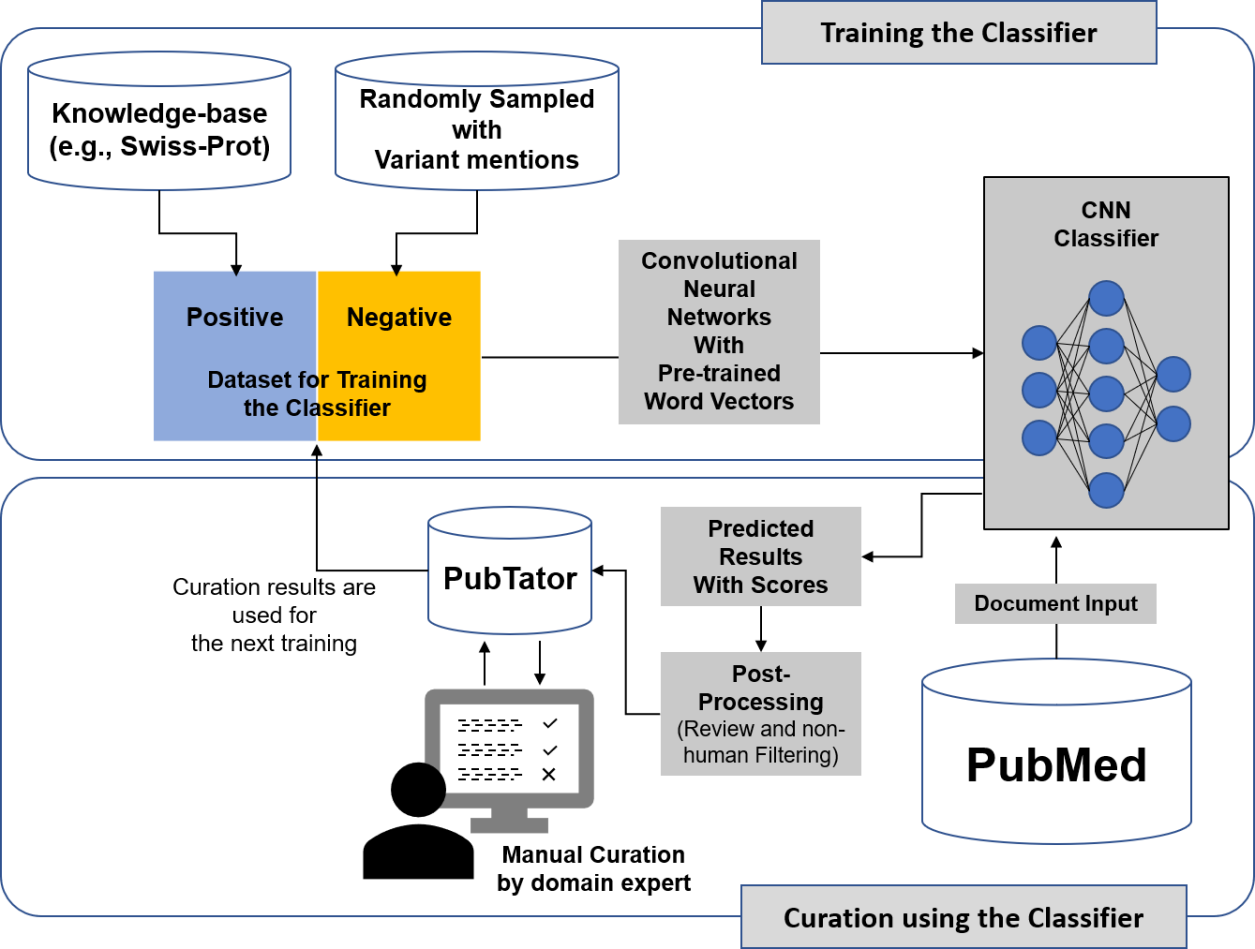
Literature triage using our deep learning framework.

Fig 1 shows the overview of our proposed framework. In a nutshell, using the previously curated publications from each knowledge base as positive examples and other variant-containing publications as negatives, we first train our machine learning classifiers. Then, we classify and rank new publications in PubMed using the trained classifier. Finally, we import classified results into PubTator, and provide the results to curators for manual verification.

For the evaluation of our method, we collected publications from UniProtKB/Swiss-Prot [1, 2] and the GWAS Catalog [3], both of which are widely used variant information knowledge bases containing several thousand manually curated publications. For method comparison, we also collected a previous document triage dataset called mycoSet [20] where other machine learning approaches were tested.

### Document classification results

We evaluated our deep learning-based triage method on three different datasets. Table 1 shows the performance of our method on the publications in UniProtKB/Swiss-Prot and the GWAS Catalog published before 2017 and mycoSet. Overall, our method achieved high performance on this triage task. We hypothesized that our method would achieve higher performance on the GWAS Catalog dataset than the other two datasets because most of the GWAS-related publications contain terms such as “GWAS” or “Genome-wide association”.

**Table 1:** Classification performance on UniProtKB, the GWAS Catalog and mycoSet. (CNN: Convolutional Neural Networks, SVM: Support Vector Machine, LMT: Logistic Model Trees).

Dataset	Methods	Precision	Recall	F1
**UniProtKB/Swiss-Prot**	Our Method (CNN)	0.913	0.934	**0.923**
	LinearSVC (SVM)	0.896	0.920	0.908
**The GWAS Catalog**	Our Method (CNN)	0.973	0.991	**0.982**
	LinearSVC (SVM)	0.965	0.980	0.972
mycoSet*	Our Method (CNN)	0.602	0.667	**0.633**
	LinearSVC (SVM)	0.566	0.627	0.595
	mycoSORT (LMT)	0.552	0.6	0.575

* In this table, the positive vs. negative ratio of mycoSet is 1:9, and that of the other datasets is 1:1.

We compared our classification results with those of the LinearSVC classifier which is a non-deep learning-based method and has achieved the best results in a recent text classification task [29]. LinearSVC is a classifier based on Support Vector Machine with a linear kernel. We used the implementation of LinearSVC with Lasso regression in Scikit-Iearn [30] for Python.

We also compared our classification results with those of mycoSORT [20]. Our method achieved higher precision, recall, and F1 scores than mycoSORT [20], which uses traditional machine learning techniques such as SVM, Naïve Bayes and Logistic Model Trees, as shown in Table 1. It is important to note that our model did not use any preprocessing, information tagging, or feature engineering steps, all of which were used in mycoSORT.

Notice that the ratio of positive vs. negative instances of mycoSet is set to 1:4 for the training and validation sets, and 1:9 for the test set in order to be consistent with Almeida et al.’s mycoSORT [20] evaluation settings. The other datasets have 1:1 ratios for their training, validation and test sets. Note that in practice, those ratios are quite different in the triage process (see details in the section on imbalanced dataset below).

### Word distribution in the datasets

To better understand the classification results, we obtained lists of the enriched words from the positively classified publications. We counted the number of times each word was mentioned in the positive and negative publications and obtained the top 100 most statistically significant words from each dataset using chi square test. Table 2 shows the 26 most significant terms in each dataset after manually combining the different forms of the same word (e.g. plural and past tense). An interesting observation is that documents in the UniProtKB/Swiss-Prot dataset often contain the word "mutation" while documents in the GWAS Catalog contain “variants” or “SNP(s)” at the top of its list. The words that overlap in the queries of each databases’ query-based triage method are highlighted.

**Table 2:** Lists of the most significant words in the positively classified publications. (The words that are used as queries in the query-based method of each database are highlighted.).

UniProtKB/Swiss-Prot		NHGRI-EBI GWAS Catalog	
mutation(s)	syndrome	wide	variants
gene	exon(s)	genome	meta
cdna	encoding	association(s)	european
human	chromosome	loci (or locus)	identify (-ies, -ied)
sequence	two	snp(s)	susceptibility
missense	region	gwas	near
families	acid	p	ancestry
novel	coding	=	chromosome
amino	domain	10	significance
identified	expressed	genetic	8
family	recessive	study	independent
autosomal	affected	replication (replicated)	conducted
protein	cloning	associated	cohorts

The different purposes of the two different knowledge bases are reflected in the differences in the word lists. Since the UniProtKB/Swiss-Prot positive publications contain protein altering mutations, its word list includes words such as “protein,” “amino,” “acid,” “sequence,” “coding,” “region,” and “missense.” Words related to typical GWAS study design and aims (cohort, replication, susceptibility and p-value related tokens such as ‘p,’ ‘ = ‘ and ‘10’) are also found.

The GWAS Catalog results contain terms such as “Genome-wide association study” or “GWAS,” which are included in the PubMed query used by the query-based method of the knowledge base. However, the UniProtKB/Swiss-Prot results do not contain the terms that are included in the query which is “functional characterization and functional analysis”.

Based on this result, we verify that our method achieves high performance in classifying publications. We also confirmed that the positively classified publications contained the correct signals.

### Ranking results of unbalanced datasets in a real-world setting

In a real-world application, we need to address the issue of extremely unbalanced datasets in terms of the ratio between positive and negative documents. Ranked results with scores are helpful in practice for manual curators because they can freely decide the number of documents to curate or discard.

**Fig 2:**
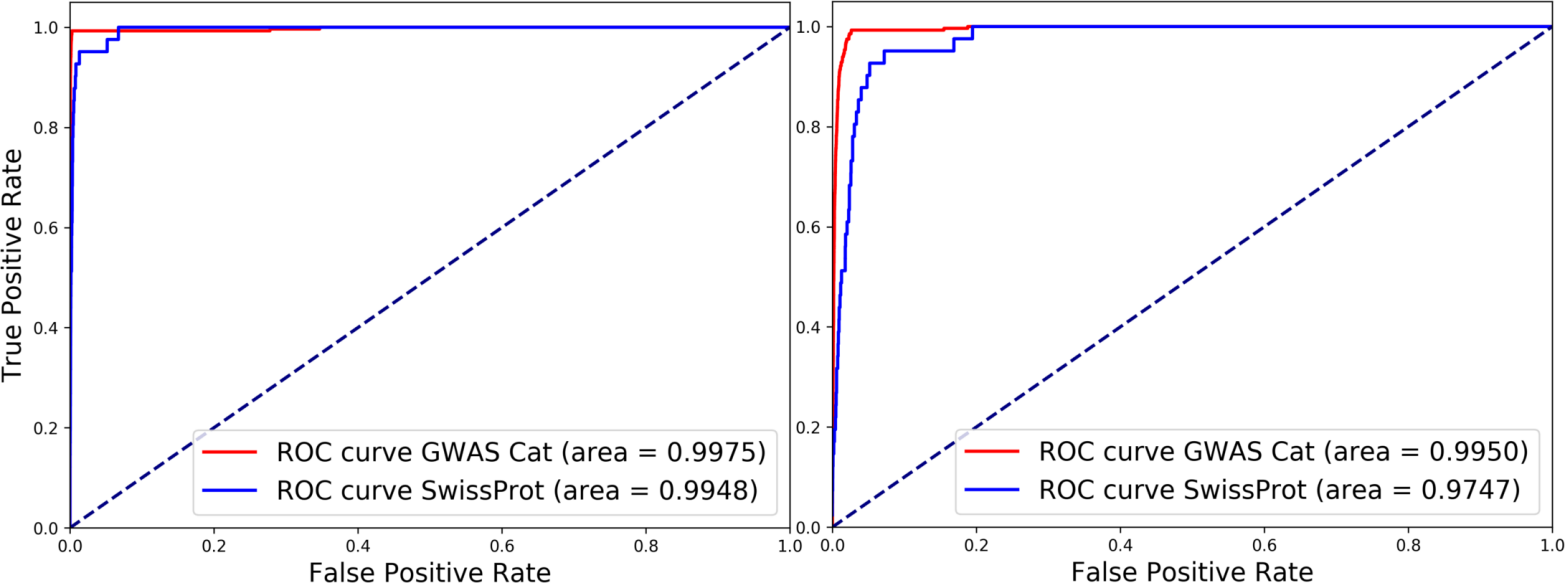
**ROC curves of the classification results on the 2017JanJul group of UniProtKB/Swiss-Prot (Blue) and the GWAS Catalog (Red)–**(a) Curves in all the publications, (b) Curves in the publications containing mutations at the abstract level.

Between January to July 2017, 23 and 225 publications were included by UniProtKB/Swiss-Prot and the GWAS Catalog through manual curation, respectively. Thus, these articles are treated as positives. The other 0.6 million publications are considered as negative. Since these datasets are extremely imbalanced, we evaluated our method in ranking/scoring using receiver operating characteristic (ROC) curves. Fig 2(A) shows the ROC curves of the ranked results by our method. We can see that most of the positive publications are ranked at the very top of the results of the classifiers and achieved high AUROCs such as 0.995 and 0.998 for UniProtKB/Swiss-Prot and the GWAS Catalog, respectively.

Additionally, we plotted Fig 2(B) using only the publications containing variants at the abstract level. We used tmVar [8, 31] to find the variant-containing publications and around 10,000 of those were found. In this result, the ROCs are slightly lower compared to those shown in Fig 2(A), but still we can see that all the positive publications are ranked in the top of the results even in variant-containing publications.

### Utility assessment

For evaluating its utility, our method was applied to the triage process of UniProtKB/Swiss-Prot and the GWAS Catalog, in collaboration with the database curators. The comparison between the query-based method and our method for both databases is provided in Table 3. To evaluate and compare our method’s results with those of the query-based method, the curators of both the databases manually curated not only the results of the query-based method but also the publications found only by our method. We consider all the curatable publications found by both methods as positives. The documents not found by either method are considered as negatives, although some might have been deemed as curatable if retrieved. Hence, the recall we reported in this section might be slightly lower than the actual recall. Nonetheless, we still believe our recall is useful for comparing the performance of the two methods.

**Table 3:** Comparison of the results of our method with those of the query-based method in the UniProtKB/Swiss-Prot and GWAS Catalog triage. Both query-based and CNN-based results were evaluated by the curators, resulting in the total number of curatable publications below.

		UniProtKB/Swiss-Prot	GWAS Catalog
**Total number of target publications**		4,680 (3 months, variant-containing publications)	64,405 (3 weeks, all publications)
**Total number of curatable publications**		424	27
**Query-based**	**Total**	79	304
	**Curatable**	36 (P: 45.57%, R: 8.49%)	27 (P: 8.88%, R: 100%)
Our CNN-based Method*	**Total**	501	98
	**Curatable**	413 (P: 82.43%, R: 97.41)	26 (P: 26.53%, R: 96.30%)

* As requested by the curators, UniProtKB results are filtered using tmVar as only articles with explicit variant mentions are within the scope of its data curation.

#### Machine learning-assisted manual curation framework—PubTator

To manage the curation process more efficiently, we used the PubTator curation system [32]. After users uploading a list of PMIDs for evaluation, PubTator provides users with an organized web-based user curation interface that displays the text of a publication in which biomedical entities are highlighted. Furthermore, users can indicate whether the publications are curatable by clicking a button. The curation results can be saved and downloaded for future use. Manual curation using PubTator has proven to be efficient in a previous study [15].

We generated the prediction results of the databases, uploaded them to PubTator, and created a collection of publications for manual curation. The curators of each database, who are also domain experts, curated the collections and sent their feedback through the PubTator system.

#### UniProtKB/Swiss-Prot

For UniProtKB/Swiss-Prot, we provided collections of positively predicted articles published in August, September and October 2017. Since the curators of UniProtKB/Swiss-Prot wanted to curate publications containing variant names at the abstract level, we used tmVar [31] to remove publications without variant mentions.

Table 3 shows the comparison of the results of our machine learning-based method and those of the current query-based UniProtKB/Swiss-Prot method. Our machine learning-based method found more publications than their current query-based method. Also, our method achieved a precision of 82.43% while the query-based method obtained a lower precision of 67.74%. Out of all the 425 curatable papers, our method missed only 12 publications (97.17% recall) compared with the query-based method that missed 398 publications (6.35% recall). Our method found 11 curatable publications that the query-based method could not find. The manual curator of UniProtKB/Swiss-Prot confirmed that our method addresses their need to get a wide set of articles with high precision without returning an overwhelming number of items.

#### The GWAS Catalog

We predicted publications for the GWAS Catalog for three weeks from January 10, 2018 to January 30, 2018. We compared our results with their query results in Table 3. Since the GWAS Catalog is not interested in non-human research and review papers, we found and removed those publications using PubTator and PubMed meta information.

As shown in Table 3, our method achieved a precision of 26.53% versus a precision of 8.88% obtained by the query-based method. Our method retrieved a smaller number of results which include all the relevant publications except for one (PMID 29313844) found by the traditional query-based method. Thus, our method returns 1/3 of the results returned by the query-based method, which reduces the number of irrelevant publications for manual review by curators. In this case, our method did not find any new publications beyond the query-based results. Also, our method missed a curatable publication that the query-based method found, which is explained in the Discussion section.

## Discussion

Our results demonstrate that our machine learning triage method works well on different datasets in different settings. Both the UniProtKB/Swiss-Prot and the GWAS Catalog manual curation teams confirmed that our method achieved higher precision than the previous query-based triage methods without significantly compromising recall. In the UniProtKB/Swiss-Prot result, our method could find many relevant publications that the query-based method could not find. In the GWAS Catalog case, our method significantly improved the efficiency of the curation process by reducing the number of papers that required review. Both results show that our method can replace the traditional query-based triage methods of manually curated databases.

However, even though our method works well and improves the efficiency of the manual curation process, it is difficult for our method to include new types of articles based on new interests. For example, the GWAS Catalog, which has collected publications related to only genome-wide array-based association studies so far, might find it difficult to find whole-exome sequencing publications since our classifier is trained on only the publications related to array-genotyped GWAS and there are no whole-exome sequencing papers in the training set. This is known as the “filter bubble” problem of personalized search, social media and news recommender systems [33]. For example, based on the behavior of a user, personalized news recommendation algorithms suggest news articles on certain topics as results. The user selects some of the results recommended by the algorithm, and the algorithm learns from those results again to recommend similar results. After this process is repeated a few times, the recommendation algorithm narrows down the recommendation results based on the interests of the user, which makes it more difficult for users to view new information other than the results recommended by the algorithm. To solve this problem, the curators need to manually find new topic publications as a separate activity. However, the curators and our team both agreed that our classification and ranking methods would save curators considerable time in standard triage. Thus they have extra time to manually find new subjects, using new queries in PubMed or using their intuition, which can solve the filter bubble problem.

The other limitation of our method comes from the “black box” property of the deep neural network models [34, 35]. In the utility assessment of the GWAS Catalog section, our method did not retrieve a publication (PMID 29313844) while the query-based method did. The confidence score of the publication obtained by our method was 0.451 which is slightly lower than the default threshold of the classifiers (0.5). We investigated why the publication was scored low even though it contains GWAS relevant terms, such as “p-value” and “GWAS,” however we could not clearly understand why this publication was not scored highly enough because deep neural networks are “black boxes.” With other traditional machine learning models such as decision tree, k-nearest neighbor and logistic regression, it is possible to see how the classification of each case is made by looking at the decision rules, neighbors, or weight of features, respectively; however, it is not possible in deep neural network models. This example shows the limitation of deep learning methods.

The hardest part of designing our method was that there is no negative gold-standard dataset available for training the classifiers. We could not collect any of the negative data for training / testing our method as we were able to for mycoSet [20]. We constructed negative datasets using randomly collected variant containing publications using tmVar after filtering out the positive publications; however, there are chances that some positive examples are included in the negative dataset. Using tmVar for generating negative dataset may also cause some bias in the classification results, because all the negative publications are tmVar positive. Our method still over-performed compared to the query-based triage methods. If we can collect enough gold-standard negative publications, our method may perform better. Since PubTator is now used as a manual literature triage framework for several knowledge bases such as UniProtKB/Swiss-Prot and the GWAS Catalog, we can easily collect negative publications that curators mark as irrelevant and we expect to use them as negative gold-standard data for training the classifiers in the future.

In this research, we focus on the triage of variant-related knowledge bases; however, our method can be applied to any knowledge base that relies on manual curation and to a triage process if it has a sufficient number of documents for training the classifier.

We also believe it would be interesting to use our method for knowledge bases of biomedical relations such as protein-protein interactions and drug-drug interactions. Although we did not use any preprocessing in our method, named-entity tagging might be helpful in these tasks.

## Materials and methods

### UniProtKB/Swiss-Prot

UniProtKB/Swiss-Prot [2] is a knowledge base of protein sequence and functional information based on manual curation and is a part of the universal protein knowledge base UniProt [1]. UniProtKB/Swiss-Prot contains rich information on genomic variants that affect protein function [2]. Poux et al. [36] explained that the curation process of UniProtKB/Swiss-Prot is expensive and time consuming. Currently, the triage process of variant information in UniProtKB/Swiss-Prot is performed using manually pre-defined queries in PubMed; however, it is difficult to generate the perfect candidate set using such queries, and to find all the relevant publications from these queries. The variant publication curator of UniProtKB/Swiss-Prot explained that they are currently curating variant publications that contain any of the following: 1) missense nucleotide substitutions resulting in amino-acid changes, 2) nonsense nucleotide substitutions producing a stop codon and resulting in protein truncation, 3) small in-frame nucleotide insertions resulting in the insertion of few amino-acids at the protein level, and 4) small in-frame nucleotide deletions resulting in the deletion of few amino-acids at the protein level. If a publication does not contain any of the above, it is marked as “TBD” (to be determined) or “not curatable.” We used UniProtKB/Swiss-Prot human data downloaded on September 20, 2017 [37].

### The NHGRI-EBI GWAS Catalog

The GWAS Catalog [3] provides manually curated genome-wide association study information from published results. The Catalog contains more than 50,000 unique SNP-trait associations from over 3,000 published research studies. The GWAS Catalog has strict guidelines for study eligibility. For example, the publications must be array-based genome-wide association studies of humans and examine >100,000 SNPs selected to tag variation across the genome. The detailed criteria of eligible publications for the GWAS Catalog are explained on their website [19], and if publications do not meet these criteria, they are not curated. All the publications need to be determined as eligible by at least two curators to be included in the knowledge base. For the triage process, the curators use general query terms to find GWAS-related publications in PubMed and manually filter the papers that do not meet the criteria. We downloaded the October 11^th^ 2017 issue of the GWAS Catalog from its website.

### Dataset construction

We collected publications from UniProtKB/Swiss-Prot [1, 2], the GWAS Catalog [3], and mycoSet [20]. Since we did not have curated gold-standard negative data from the knowledge bases, we generated them de novo: we considered an article negative if it was not curated in the knowledge bases, but has one or more variant mentions (found by tmVar). From all the PubMed abstracts and PMC full-text articles with open access, we collected more than 442,000 articles containing variants using tmVar [8, 31].

Using these publications as a gold-standard, we trained classifiers for each knowledge base. We downloaded only PMIDs from each data source. Given a PMID, we used the NCBI Entrez Programming Utilities [38] to collect its title and abstract, journal information, and publication type.

Based on the date (Entrez Date (EDAT)) of each publication, we organized the publications in UniProtKB/Swiss-Prot and the GWAS Catalog into the following three groups: (1) the Before2017 group, which contains articles published before 2017, (2) the JanJul2017 group, which consists of articles published from January 2017 to July 2017, and (3) the AfterAug2017 group, containing articles published after August 2017. We used the papers in the Before2017 group of each knowledge base to design, train, and evaluate the performance of our method in binary classification and used papers in the JanJul2017 group for evaluating the ranking function of the method for curation. We divided the data in this way because we can train only on the past data to predict newly published data in a real-world setting. This data separation setting was used for simulating the real-world triage process. For the Before2017 group of each knowledge base, we randomly collected the same number of negative publications from the negative dataset that are excluded in each positive dataset.

### mycoSORT and its dataset—MycoSet

Almeida et al. [20] constructed a dataset and used machine learning methods for a triage task. Their dataset called mycoSet is manually curated and contains publications on enzyme family information. It contains a total of 7,583 PMIDs, of which 9.88% are positive. Their machine learning method used not only the abstracts and titles of the publications, but also used the additional enzyme information (Enzyme Commission numbers and the “RegistryNumber”) tagged to the publications. They also performed feature extraction using handcrafted rules, and preprocessed the text using another text mining system called mycoMINE. Naïve Bayes, Logistic model trees, and support vector machine (SVM) were used to classify the publications. In [20], a total of 108 classification results of mycoSORT are listed (three different machine learning classifiers using a total of 36 different ratios of positive and negative publications in the training set), and the logistic model tree classifier with a negative-positive ratio of 4:1 achieved the best F1 score of 0.575.

### Convolutional neural networks (CNN)

We used convolutional neural networks as our machine learning classifiers. CNN is a deep learning method that uses feed-forward multi-layer neural networks such as fully-connected layers, pooling layers and convolutional layers with shared weights for entire inputs [39]. Although CNNs were typically used for image-related works in previous research [40–42], they have recently achieved good results in text related works [22–24, 27, 43]. In addition, it does not require labor intensive feature engineering by domain experts. Hence, our CNN-based approach can be applied to datasets from different databases. We trained three different CNN classifiers on three different sets of PMIDs collected from UniProtKB/Swiss-Prot, the GWAS Catalog and mycoSet, respectively. The details of the datasets are provided in Table 4. The positive to negative ratio of the publications is balanced in all the training, testing and validation sets of UniProtKB/Swiss-Prot and the GWAS Catalog by generating the same amount of negative data.

**Table 4:** Statistics of the datasets.

	UniProtKB/Swiss-Prot	The NHGRI-EBI GWAS Catalog	mycoSet Positive	mycoSet Negative
Version	Sep. 20, 2017	Oct. 11, 2017 Ver 1.0.1 Studies	-	-
Total # of PMIDs	12,779	3,164	749	6,902
PMIDs with abstracts	11,978	3,143	746	6,575
**EDAT before 2017**	**11,955**	**2,892**	N/A	N/A
**EDAT** **from Jan to July 2017**	**23**	**225**	N/A	N/A

We built the CNN text classification codes using Keras [44] and TensorFlow [45], based on the implementation [22] available at https://github.com/yoonkim/CNN_sentence. We used the 200-dimensional word2vec vectors of Pyysalo et al. [46–48], which were pre-trained on all the PubMed abstracts and PubMed Central open access full texts, available at http://bio.nlplab.org/. The windows of the filters were set to 3, 4, and 5. A dropout rate of 0.5, a learning-rate of 0.5*10^−5^, and a mini-batch size of 50 were used. We followed all the other detailed options as Kim suggested. We used the same parameter settings for all the three classifiers, and we used GeForce GTX 1080 Ti GPU on Linux CentOS 7.4 to train the models. Instead of intensive parameter settings, we used most of the same parameters used in the previous implementation. Our modified source codes and the detailed settings are available at https://github.com/ncbi-nlp/VarTriage.

### Assessment methods

We used precision, recall and F1-score to evaluate our classification methods. The scores are calculated as follows:

Precision=(TruePositive)/(TruePositive+FalsePositive)

Recall=(TruePositive)/(TruePositive+FalseNegative)

F1‑score=(2×Precision×Recall)/(Precision+Recall)

We randomly divided each dataset into 10 folds and used 8 of them for training, 1 for validation and 1 for obtaining the final results which are provided in Table 1. The test sets are used only to obtain the final scores; they are not used for setting parameters or selecting models.

We also used receiver operating characteristic (ROC) curves to evaluate the ranking performance of our methods. ROC curves are plotted using true positive rate (Y axis) and false positive rate (X axis). When the true-positive publications obtain higher scores and true-negative publications obtain lower scores, the area under curve (AUC) becomes larger, which means that the method is ranking the items accurately.

## Supporting information

S1 Text PubMed queries for the query-based methods, and the URLs for the data downloads. (DOCX)
