# Supplementary material for: Scaling up data curation using deep learning: An application to literature triage in genomic variation resources
Source: PLoS Comput Biol. 2018 Aug 13;14(8):e1006390. doi: 10.1371/journal.pcbi.1006390 (PMC6107285; doi:10.1371/journal.pcbi.1006390)
Supplement: S1 Text — (DOCX) [file pcbi.1006390.s001.docx]

**S1 Text. Supporting Information**

**PubMed Queries for the query-based methods**

**Query of UniProtKB/Swiss-Prot**: (((mutations) AND "functional characterization"[TITLE/ABSTRACT])) OR ((mutations) AND "functional analysis"[TITLE/ABSTRACT])

**Query for the GWAS Catalog**: ((genomewide[All Fields] OR (("genome"[MeSH Terms] OR "genome"[All Fields]) AND wide[All Fields]) OR genome-wide[All Fields] OR ("genome-wide association study"[MeSH Terms] OR ("genome-wide"[All Fields] AND "association"[All Fields] AND "study"[All Fields]) OR "genome-wide association study"[All Fields] OR "gwas"[All Fields])) NOT Review[ptyp]) AND ("2018/01/10”[EDAT] : "2018/01/30”[EDAT])

**Used Data URLs**

**Downloaded data URL for UniProtKB/Swiss-Prot**: ‘uniprot_sprot_human.dat’ at <ftp://ftp.uniprot.org/pub/databases/uniprot/current_release/knowledgebase/taxonomic_divisions>

**Downloaded data URL for the GWAS Catalog**: ‘gwas_catalog_v1.0.1-studies_r2017-10-10.tsv’ at <https://www.ebi.ac.uk/gwas/docs/file-downloads>

**Venn-diagram to support Table 3**

Comparison of results of our method with those of the query-based method in UniProtKB/Swiss-Prot and the GWAS Catalog triage


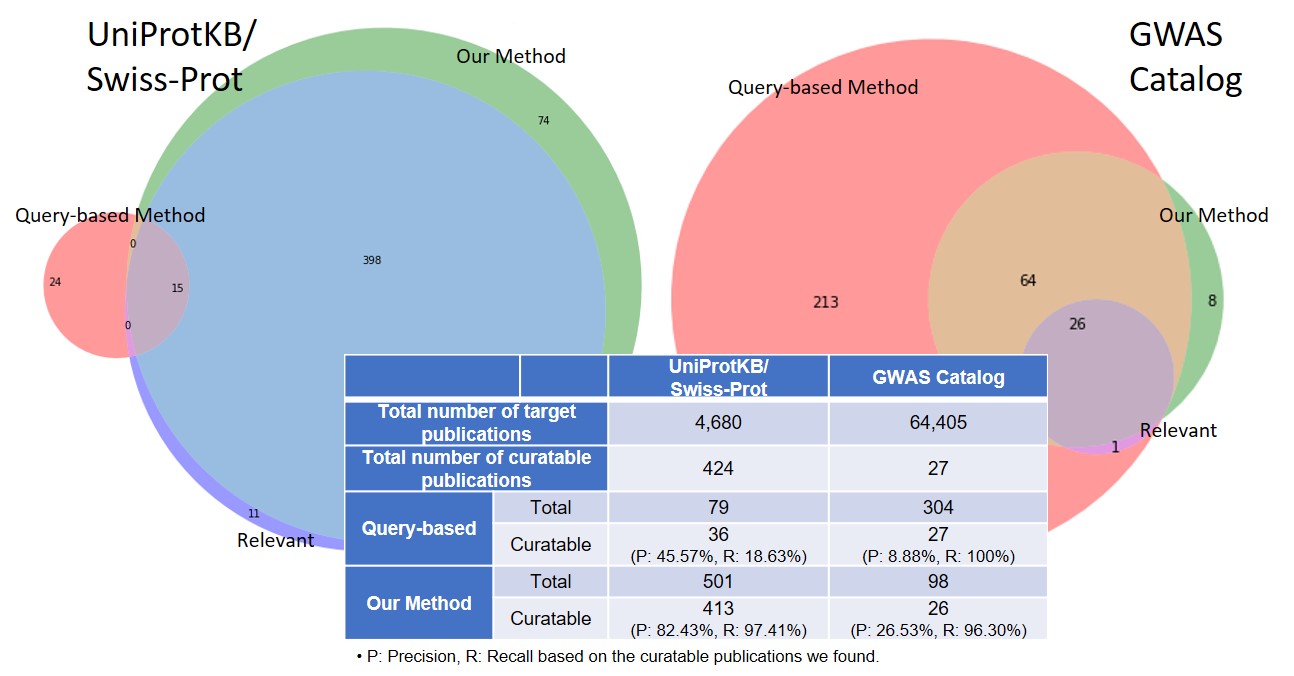


**Precision-Recall curve of the classification results on the 2017JanJul group of the GWAS Catalog** (We cannot plot the curve for UniProtKB results because of the curation for the period is still in progress at the time of the manuscript submission.)

**
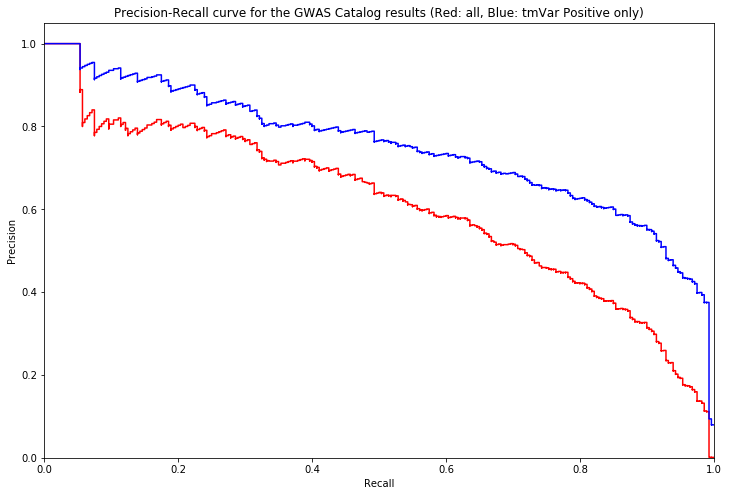
**

**Validation – Test scores of our method**

|  | **Validation** | | | **Test** | | |
| --- | --- | --- | --- | --- | --- | --- |
| **Dataset** | **Precision** | **Recall** | **F1** | **Precision** | **Recall** | **F1** |
| **UniProtKB /Swiss-Prot** | 0.920 | 0.936 | 0.928 | 0.913 | 0.934 | **0.923** |
| **The GWAS Catalog** | 0.988 | 0.988 | 0.988 | 0.973 | 0.991 | **0.982** |
| **mycoSet** | 0.745 | 0.622 | **0.679** | 0.602 | 0.667 | **0.633** |

* Positive : Negative ratios of the training/validation datasets and test datasets of mycoSet are set to 1:4 and 1:9, respectively which is same as Almeida et al’s evaluation settings. For other datasets have 1:1 ratios for training, validation and test datasets. Those ratios are not the same in the actual triage environment.
